# Dissolution Behavior of Mineral Elements in Dahongpao Tea, Its Association with Flavor Quality, and Potential Contribution of Infusions to Dietary Reference Intakes

**DOI:** 10.3390/foods15173119

**Published:** 2026-09-02

**Authors:** Miao Jia, Miaoen Qiu, Yuhua Wang, Aiqi Wang, Xiaoli Jia, Jianghua Ye, Haibin Wang, Zeyan Wu

**Affiliations:** 1 College of Life Science, Fujian Agriculture and Forestry University, Fuzhou 350002, China; jiam202309@outlook.com (M.J.); miaoenqiu06@outlook.com (M.Q.); 22427025012@fafu.edu.cn (Y.W.); 2 College of Life Science, Longyan University, Longyan 364012, China; aiqiwang2004@outlook.com; 3 Fujian Key Laboratory of Big Data Application and Intellectualization for Tea Industry, College of Tea and Food Science, Wuyi University, Wuyishan 354300, China; jiaxl2010@wuyiu.edu.cn (X.J.); jhye1998@wuyiu.edu.cn (J.Y.)

**Keywords:** Dahongpao tea, mineral elements, volatile profile, taste characteristics, suitable tea consumption

## Abstract

Mineral elements affect tea nutritional and flavor quality, but systematic research on their dissolution behavior and links to volatile and taste profiles in Dahongpao tea is lacking, as is evidence regarding contribution to mineral intake for different populations. In this study, nine Dahongpao teas were brewed five times. Eleven elements were measured by inductively coupled plasma mass spectrometry (ICP-MS) with first-order kinetic fitting; volatile profiles were analyzed by electronic nose and taste attributes by electronic tongue; the contribution of tea infusions to dietary reference intakes was estimated from Chinese Dietary Reference Intakes (2023). The results showed that electronic nose analysis revealed distinct variation in volatile compound classes among the nine samples, with broad range and sulfur–organic compounds showing the highest response intensities. Electronic tongue analysis showed clear differences in taste profiles, particularly in astringency, umami, and sweetness, across the nine samples. Regarding dissolution behavior, Mg had the highest dissolution rate (67.24–70.94%), while Fe, Ca, K, and Zn were below 10%; dissolution followed first-order kinetics and total dissolved amounts correlated with total contents except for Fe. Network analysis integrating element data with volatile and taste profiles demonstrated that Mg and Mo contents were positively correlated with multiple volatile classes (e.g., sulfur–organic, broad range), while Ca, Mg, and Fe dissolution rates significantly correlated with richness and aftertaste-A, and Mo inversely correlated with sourness and saltiness. Contribution assessment showed that only Ca, K, Mg, and Se could be marginally supplemented, but the tea amounts needed far exceeded typical intake. In summary, element dissolution in Dahongpao tea is co-regulated by brew count and total content, with complex element–flavor interactions. Tea is not a primary mineral source; consumption should be individualized by age and physiological status.

## 1. Introduction

As one of the most popular beverages worldwide, tea has long received extensive attention for its quality characteristics and health benefits. Wuyi rock tea, produced in Wuyishan, Fujian Province, China, belongs to the oolong tea category and is renowned for its unique “Yan Yun” and mellow taste [1]. Among the most representative varieties of Wuyi rock tea, Dahongpao is highly favored by consumers for its rich and layered aroma as well as its mellow and sweet aftertaste [2]. However, the sensory quality of tea is influenced by multiple factors, including tea plant cultivar, growing environment, processing technology, and brewing method [3,4,5]. Recent evidence has demonstrated that mineral elements not only participate in the regulation of tea plant growth, development, and secondary metabolite biosynthesis but may also influence the taste characteristics and aroma profiles of tea infusions by affecting the release and interactions of chemical components in the tea liquor [6,7].

Mineral elements are important components of tea, and their contents and chemical forms directly affect the nutritional value and sensory quality of tea. To date, various elements such as K, Ca, Mg, Fe, Zn, Cu, Mn, Se, Cr, and Mo have been detected in tea, and their contents are significantly regulated by soil background, fertilization management, harvesting season, and processing technology [8,9,10]. However, elements in tea can only be ingested by the human body after being dissolved into the tea infusion during brewing. Therefore, clarifying the dissolution behavior of elements during multiple brews is a prerequisite for assessing the actual nutritional contribution of tea [11]. Previous studies have consistently shown that element contents vary among tea types, with K, Ca, and Mg being the most abundant, and dissolution rates generally decrease with increasing brew number, with the first brew contributing the majority of total dissolution [12,13]. In addition, significant differences in elemental dissolution rates have been observed among different teas during steeping, which are closely related to tea variety, raw material, and processing method [14]. As a representative of Wuyi rock tea, Dahongpao, with its unique roasting process and compact appearance, may exhibit elemental dissolution behavior distinct from that of other tea types.

Mineral elements, as important components of tea, not only determine the nutritional composition of the tea infusion through dissolution, but may also participate in the formation of tea flavor via multiple pathways. For instance, they or their complexes with polyphenols and organic acids can directly modify basic taste sensations (sourness, sweetness, bitterness, astringency, saltiness) and complex mouthfeel attributes such as richness and aftertaste [15]. Studies have shown that different tea types exhibit distinct dissolution patterns and sensory characteristics [16], and specific ions such as Ca^2+^ can enhance astringency while reducing bitterness, sweetness, and umami by modulating the flavor characteristics of taste-active compounds rather than by altering their solubility [17]. Moreover, synergistic interactions among multiple ions have been found to influence the physicochemical properties and sensory quality of tea infusions [18]. Collectively, these findings indicate that differences in element contents and dissolution rates are important factors affecting tea flavor quality.

Mineral elements in tea are ingested primarily through brewed infusions, and their requirements vary significantly among populations of different ages and physiological statuses [19,20]. The Dietary Reference Intakes for Chinese Residents (2023 edition) has specified adequate intakes for all age groups as well as for pregnant and lactating women [21]. However, the extent to which tea can meet these requirements and whether tea contributes meaningfully to mineral requirements among different populations remain unclear. Despite the above findings, systematic studies on multi-element analysis, dissolution behavior during multiple brews, element–flavor interactions, and population-based evaluation of the contribution of Dahongpao tea infusions to dietary reference intakes remain scarce. Accordingly, in this study, nine Dahongpao finished tea products from the Wuyishan production area in Fujian Province, China, were used as research materials. The total contents of 11 mineral elements (Ca, K, Na, Mg, Fe, Zn, Cu, Mn, Se, Cr, and Mo) in tea and their dissolution amounts during five consecutive brews were determined by inductively coupled plasma mass spectrometry (ICP-MS). A first-order kinetic model was employed to fit the cumulative dissolution patterns of the elements. Combined with electronic nose and electronic tongue techniques, the volatile profiles and taste characteristics of tea samples were obtained, and interaction network analysis was conducted to reveal the potential associations among element contents, dissolution rates, and flavor attributes. With reference to the 2023 edition of the Dietary Reference Intakes for Chinese Residents, the contribution of tea infusions to dietary reference intakes for different age groups as well as for pregnant and lactating women was assessed. This study aims to systematically elucidate the release behavior of mineral elements in Dahongpao tea and their associations with flavor quality, and to provide a quantitative basis for evaluating the nutritional contribution of tea among different populations.

## 2. Materials and Methods

### 2.1. Collection of Tea Samples and Preparation of Tea Infusions

**Figure 1:**
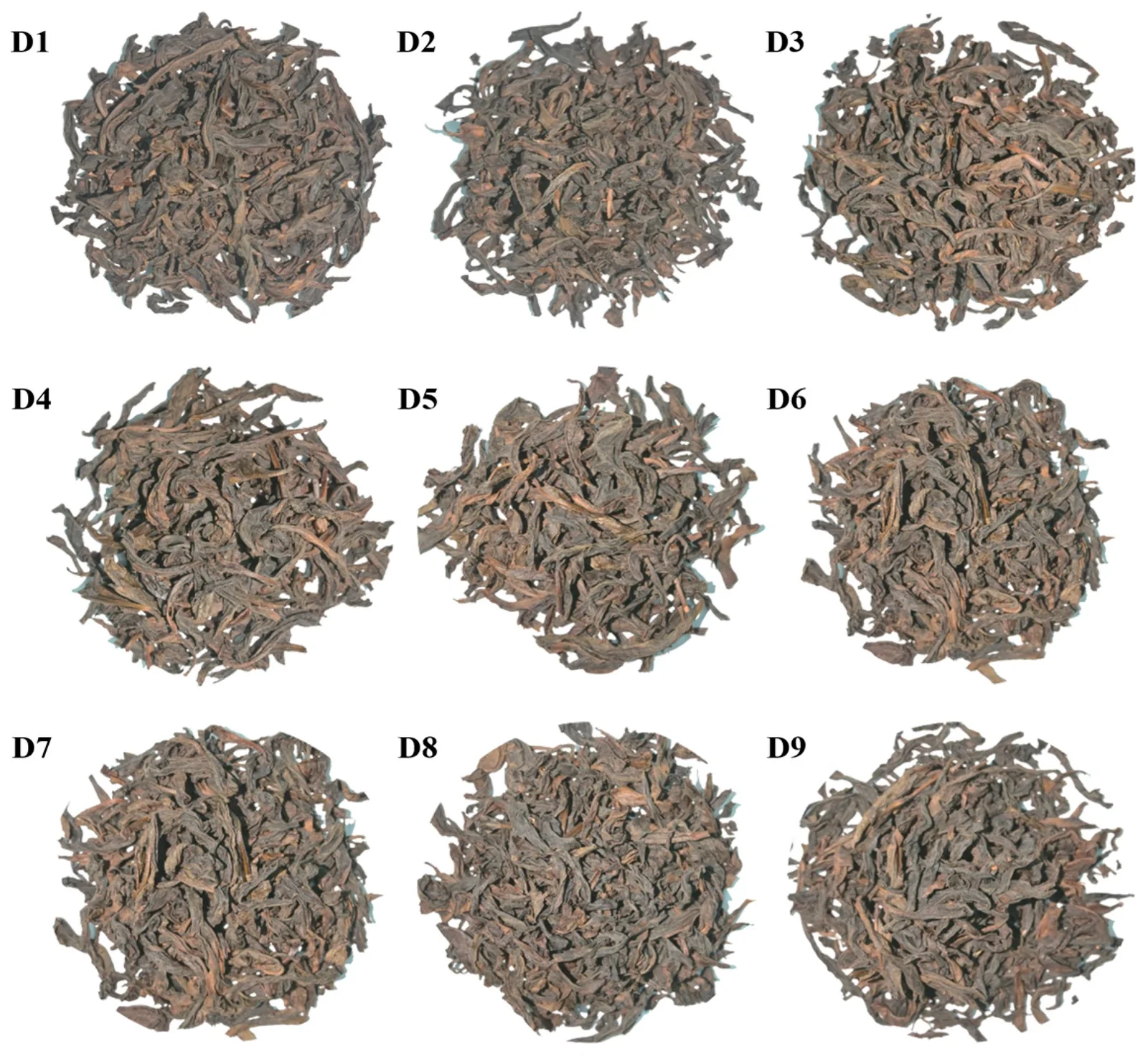
Nine Dahongpao finished tea samples. D1–D9 denote nine different Dahongpao finished tea products.

Collection of tea samples: In October 2025, nine Dahongpao finished tea samples (approximately 500 g each) were collected from Wuyishan Hexiang Tea Industry Co., Ltd., Wuyishan, China, with three independent replicates for each sample, and were labeled D1–D9 (Figure 1). The fresh tea leaves used for all nine samples were harvested in May 2025 and processed at Wuyishan Hexiang Tea Industry Co., Ltd. using identical post-harvest procedures, including withering, fermentation, kneading, and drying, to ensure that processing conditions were consistent across all samples. The samples were intentionally selected to represent different geographical origins within the Wuyishan tea region: D1, D2, and D3 were produced from fresh leaves sourced from three separate tea plantations in one area, with pairwise distances exceeding 5 km; D4, D5, and D6 were produced from three tea plantations in a second area, with distances greater than 3 km; and D7, D8, and D9 were produced from three tea plantations in a third area, with distances greater than 2 km. This sampling design allowed for the assessment of elemental composition variability among tea samples from different production areas. For homogenization, each of the nine tea samples were thoroughly mixed and divided into three portions using the quartering method. One portion was ground to a fine powder and passed through a 100-mesh nylon sieve; this powdered material was used for the determination of element contents in tea leaves. The second portion was retained in its original intact leaf form and used for the preparation of tea infusions, as described below, to simulate actual consumption conditions. The third portion was finely divided (without grinding into powder) and used for electronic nose and electronic tongue analyses to preserve the volatile and taste characteristics. All samples were stored in sealed polyethylene bags at room temperature (20–25 °C) in a desiccator prior to analysis.

**Figure 2:**
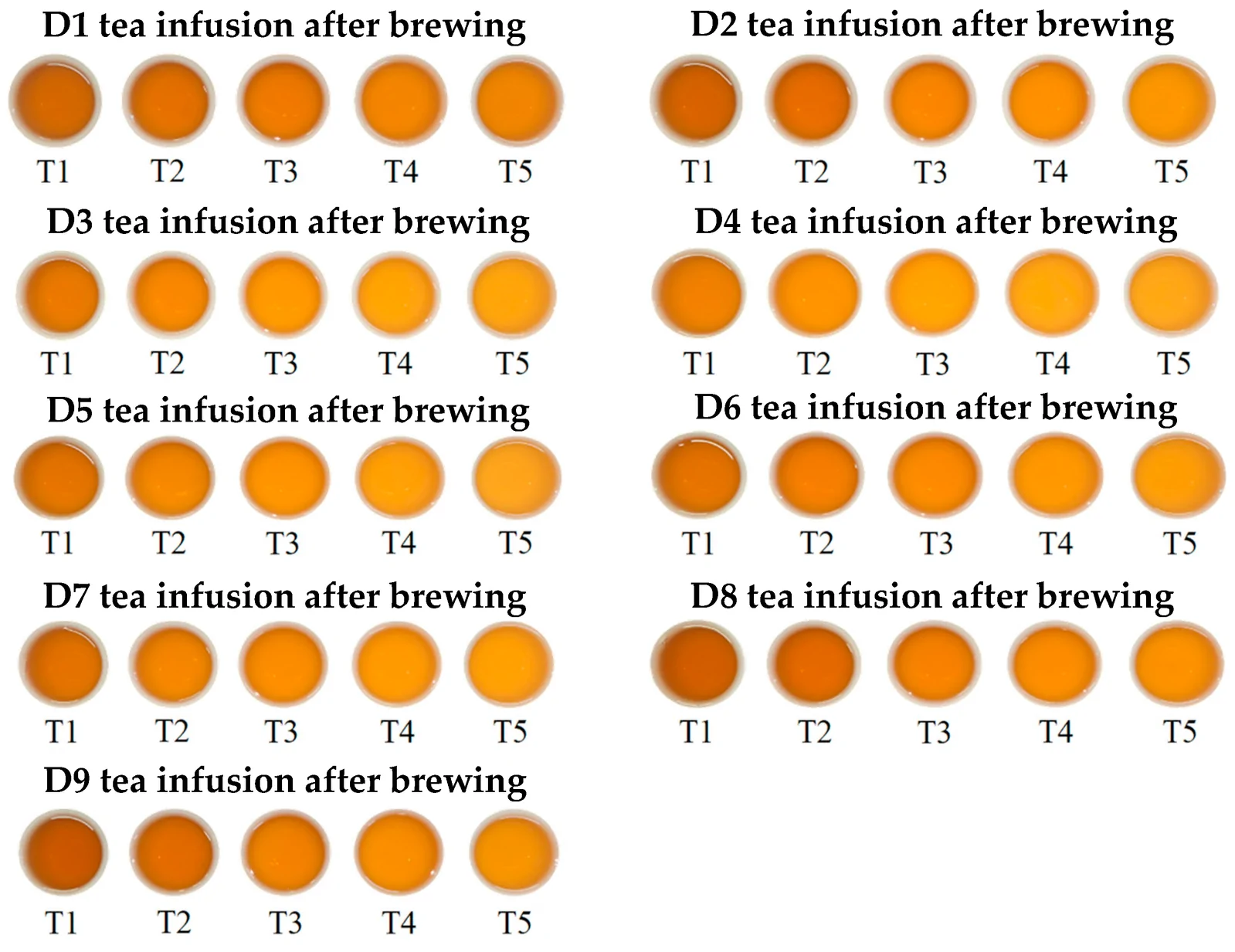
Tea infusions of nine Dahongpao finished tea samples after five consecutive brews. D1–D9 denote nine different Dahongpao finished tea products. T1–T5 denote the tea infusions of the Dahongpao finished tea samples from the first to the fifth brew, respectively.

Preparation of tea infusions: Following the actual brewing method for traditional Wuyi rock tea consumption, 8.0 g of tea sample was weighed and infused with ultrapure water (prepared using a Milli-Q Integral system (Millipore, Billerica, MA, USA), resistivity 18.2 MΩ·cm at 25 °C) at a tea to water ratio of 1:15 (*w*/*v*) and a constant water temperature of 95 °C. Reagent blanks were prepared using the same water and subjected to identical digestion and analytical procedures as the tea infusion samples, and the measured elemental concentrations in the blanks were subtracted from all sample values. Under these constant conditions, a fixed 8.0 g of tea was subjected to five consecutive infusions to simulate the actual brewing process (Figure 2). The brewing time for each infusion was set as follows: 5 s for the first infusion, and then increased by 3 s for each subsequent infusion, i.e., 8 s for the second, 11 s for the third, 14 s for the fourth, and 17 s for the fifth infusion. The infusions from the first to the fifth brew were designated T1–T5, respectively. The prepared tea infusions were subsequently used for the determination of element contents in the infusions.

### 2.2. Chemicals and Instruments

The nitric acid and hydrogen peroxide used in this study were of guaranteed reagent grade and purchased from Sinopharm Group Co., Ltd. (Beijing, China). Ultrapure water was prepared using a Milli-Q Integral system (Millipore, Billerica, MA, USA). Single-element standard stock solutions of Ca, K, Na, Mg, Fe, Zn, Cu, Mn, Se, Cr, and Mo were purchased from the National Analysis and Testing Center for Nonferrous Metals and Electronic Materials (Beijing, China). The main instruments used in this study included an electronic nose (PEN3, Air sense Analytics GmbH, Schwerin, Germany), an electronic tongue (SA402BPlus, Insent Intelligent Sensor Technology, Inc., Kanagawa, Japan), a microwave digestion system (CEM MARS 6, CEM Corporation, Matthews, NC, USA), and an inductively coupled plasma mass spectrometer (ICP-MS, Agilent 7900, Agilent Technologies, Santa Clara, CA, USA).

### 2.3. Determination of Volatile Profiles in Tea by Electronic Nose

The volatile profiles of tea samples were determined using an electronic nose, following the method described by Liu et al. [22] with minor modifications. The instrument is equipped with nine metal oxide semiconductor sensors, each exhibiting different sensitivity to various classes of volatile organic compounds. The sensors respond to the presence of volatiles by changes in electrical resistance, and the measured signals are expressed as relative resistance ratios (G/G_0_), which are dimensionless values representing the ratio of sensor resistance upon exposure to the sample gas (G) to that in the reference gas (G_0_). The sensor array detects a broad range of volatile classes, including arom–aliph, aromatic, broad-alcohol, broad-methane, broad range, hydrogen, methane–aliph, sulfur–organic, and sulf–chlor compounds. Briefly, 0.2 g of ground tea powder was weighed and placed into a 50 mL headspace vial, to which 20 mL of boiling water was added, and the vial was immediately sealed. The vial was allowed to stand at room temperature for 30 min to ensure equilibration of the headspace gas, after which the measurement was performed. The measurement time was 90 s, with a pre-sampling time of 5 s and a gas flow rate of 400 mL/min. Each sample was measured in triplicate and the average value was taken, with three independent biological replicates for each sample.

### 2.4. Determination of Taste Characteristics of Tea by Electronic Tongue

The taste characteristics of tea samples were determined using an electronic tongue, following the method described by Zan et al. [23] with minor modifications. The instrument is based on potentiometric measurement using lipid-membrane sensors that mimic the human taste reception mechanism. The sensors generate electric potential differences upon interaction with taste-active substances, and the measured output is expressed in millivolts. The sensor array comprises nine working electrodes with different lipid/polymer membrane compositions, providing selective sensitivity to sourness, bitterness, astringency, aftertaste-B, aftertaste-A, umami, richness, saltiness, and sweetness. Briefly, 1.2 g of tea sample was weighed and infused with 60 mL of boiling purified water at a tea-to-water ratio of 1:50 (*w*/*v*). The mixture was shaken and allowed to stand for 5 min. Subsequently, 25 mL of the supernatant infusion was pipetted into a 250 mL volumetric flask and made up to volume with purified water. The diluted infusion was allowed to cool to room temperature, and then 40 mL of the solution was taken for electronic tongue analysis. Each sample was measured in triplicate and the average value was taken, with three independent biological replicates for each sample.

### 2.5. Determination of Contents of 11 Elements in Tea and Tea Infusions by ICP-MS

This study aimed to determine the content of 11 elements, namely Ca, K, Na, Mg, Fe, Zn, Cu, Mn, Se, Cr, and Mo, in both tea and tea infusions.

Pretreatment of tea samples: Tea samples were dried at 60 °C in an oven for 12 h to constant weight, then ground and passed through a 100-mesh nylon sieve. Accurately, 0.5 g of powder was weighed into a digestion vessel, to which 8 mL of HNO_3_ and 2 mL of H_2_O_2_ were added. The vessel was tightly capped and subjected to microwave digestion. The digestion program was set as follows: ramp to 130 °C in 5 min and hold for 5 min; ramp to 160 °C in 5 min and hold for 5 min; ramp to 200 °C in 5 min and hold for 20 min; the microwave power was set at 1500 W. After digestion, the vessel was cooled to below 40 °C, and the digest solution was transferred to a 50 mL volumetric flask. The inner wall of the digestion vessel was rinsed three times with ultrapure water, and the solution was made up to volume and mixed well. Three independent biological replicates were prepared for each sample. Meanwhile, reagent blanks were prepared using the same procedure.

Pretreatment of tea infusion samples: An accurately measured 10 mL of the original tea infusion was transferred to a digestion vessel and evaporated to near dryness on a hot plate at 90 °C. After cooling, 6 mL of HNO_3_ and 1 mL of H_2_O_2_ were added, and the sample was digested following the same microwave digestion program as described above. The digest was transferred to a 25 mL volumetric flask and made up to volume. Three independent biological replicates were prepared for each sample. Reagent blanks were also prepared using 10 mL of the water used for tea brewing, following the same procedure.

Preparation of standard solutions: Single-element standard stock solutions (1 g/L) were stepwise diluted with 2% HNO_3_ to prepare a mixed intermediate standard solution containing all 11 elements at a concentration of 10 mg/L each. Then, six concentration levels of mixed working standard solutions were prepared by diluting the intermediate solution with 2% HNO_3_. All standard solutions were freshly prepared before use.

ICP-MS measurement: Elemental analysis was performed using an ICP-MS instrument equipped with a nickel sampler cone, a nickel skimmer cone, and an octopole collision/reaction cell. The nebulizer was a MicroMist glass concentric nebulizer, and the spray chamber was a quartz dual-pass cyclonic spray chamber. The internal standard solution was mixed online with the sample solution through a three-way valve at a flow ratio of 1:1. Tuning solution (Li, Y, Ce, Tl) was used to optimize the ion lens voltage, mass axis, and resolution to achieve sensitivities of Li > 3000 cps/ppb, Y > 10,000 cps/ppb, Tl > 8000 cps/ppb, oxide ratio (CeO^+^/Ce^+^) < 3%, and doubly charged ion ratio (Ce^2+^/Ce^+^) < 1.5%. The optimized operating parameters were RF power 1550 W, plasma gas (Ar) flow rate 15.0 L/min, auxiliary gas 1.0 L/min, nebulizer gas 0.95 L/min, sampling depth 8.0 mm, collision cell He flow rate 4.5 mL/min, scan mode peak hopping, and dwell time 50 ms per mass. Each sample was measured in triplicate. The internal standards used for each element were as follows: Sc was used as the internal standard for Na, Mg, K, and Ca; Ge for Cr, Mn, Fe, Cu, and Zn; and Rh for Se and Mo.

Method validation and quality control: The method detection limit (MDL) was calculated as three times the standard deviation of 10 blank measurements multiplied by the ratio of dilution volume to sample mass/volume, and the limit of quantification (LOQ) was defined as ten times the standard deviation multiplied by the same dilution factor. Precision was assessed by analyzing the same sample six consecutive times, and the relative standard deviation (RSD) was required to be ≤5%. Spike recoveries for sample extraction were required to be within the range of 80–120%. After every 10 sample measurements, a standard point (200 μg/L mixed standard solution) and a blank were re-analyzed; recalibration was performed if the relative deviation exceeded 10%. In addition, if the internal standard signal drift of any sample exceeded 30% of that at initial calibration, the instrument was stopped for cleaning of the nebulizer, spray chamber, and cones. After measuring high concentration samples, the rinse time was extended to 90 s, and a blank check was inserted. All laboratory wares were soaked in acid and rinsed with ultrapure water before use.

### 2.6. Assessment of Appropriate Daily Tea Consumption for Humans

Based on the data of average daily intakes of the 11 elements from the daily diet of Chinese residents (Table S1) [10,24], with reference to the adequate intakes for each element as specified in the 2023 edition of the Chinese Dietary Reference Intakes (Tables S2–S4) [21], and combined with the total amount of each element released from tea after five consecutive brews determined in this study, the appropriate daily tea consumption for humans was assessed. The calculation was performed using the following equation shown in Equation (1).

(1) ATEA_i_ = (AI_i_ − DI_i_)/TEC_i_

In this equation, *i* denotes a specific element; ATEA_i_ represents the appropriate daily tea consumption for element (g/d); AI_i_ represents the adequate daily intake of element for humans (mg/d or μg/d); DI_i_ represents the average daily intake of element from the diet (mg/d or μg/d); and TEC_i_ represents the total dissolved amount of the element in tea after 5 consecutive brews (mg/g). If (AI_i_ − DI_i_) yielded a positive value, it indicated that the element could be supplemented through tea consumption; if the value was negative, it suggested that no supplementation of that element through tea was necessary.

### 2.7. Statistical Analysis

All data were expressed as the mean ± standard deviation (SD) from three independent biological replicates. Correlation network analysis among element contents, dissolution rates, volatile components, and taste attributes was performed using Pearson’s correlation test, with significant correlations defined as *p* < 0.05. First-order kinetic model fitting was conducted using nonlinear regression analysis to characterize the cumulative dissolution patterns of elements over brewing time. The model equation was expressed as Equation (2).

(2) Y = C × (1 − e^(−k × t)^)

where Y is the cumulative release rate of a given element at brewing time t (%), C is the estimated equilibrium release rate (%) representing the maximum extractable fraction, and k is the rate constant (min^−1^) reflecting the release rate. The fitting procedure was performed using the Levenberg–Marquardt algorithm for parameter estimation, with initial values for C and k set based on the observed maximum release rate (%) and the slope of the initial linear phase, respectively. Convergence was considered achieved when the relative change in residual sum of squares between successive iterations was less than 10^−6^. Preliminary data organization was performed using Excel 2022, and all statistical analyses and data visualization were carried out using RStudio software (v4.2.3) with the following packages: ggplot2 (v4.0.1) for scatter linear regression plots and general visualization, minpack.lm (v1.2.4) combined with ggplot2 for first-order kinetic curve fitting, and fmsb (v0.7.6) with scales (v1.4.0) for radar plots.

## 3. Results

### 3.1. Analysis of Volatile Profiles in Different Tea Samples

**Figure 3:**
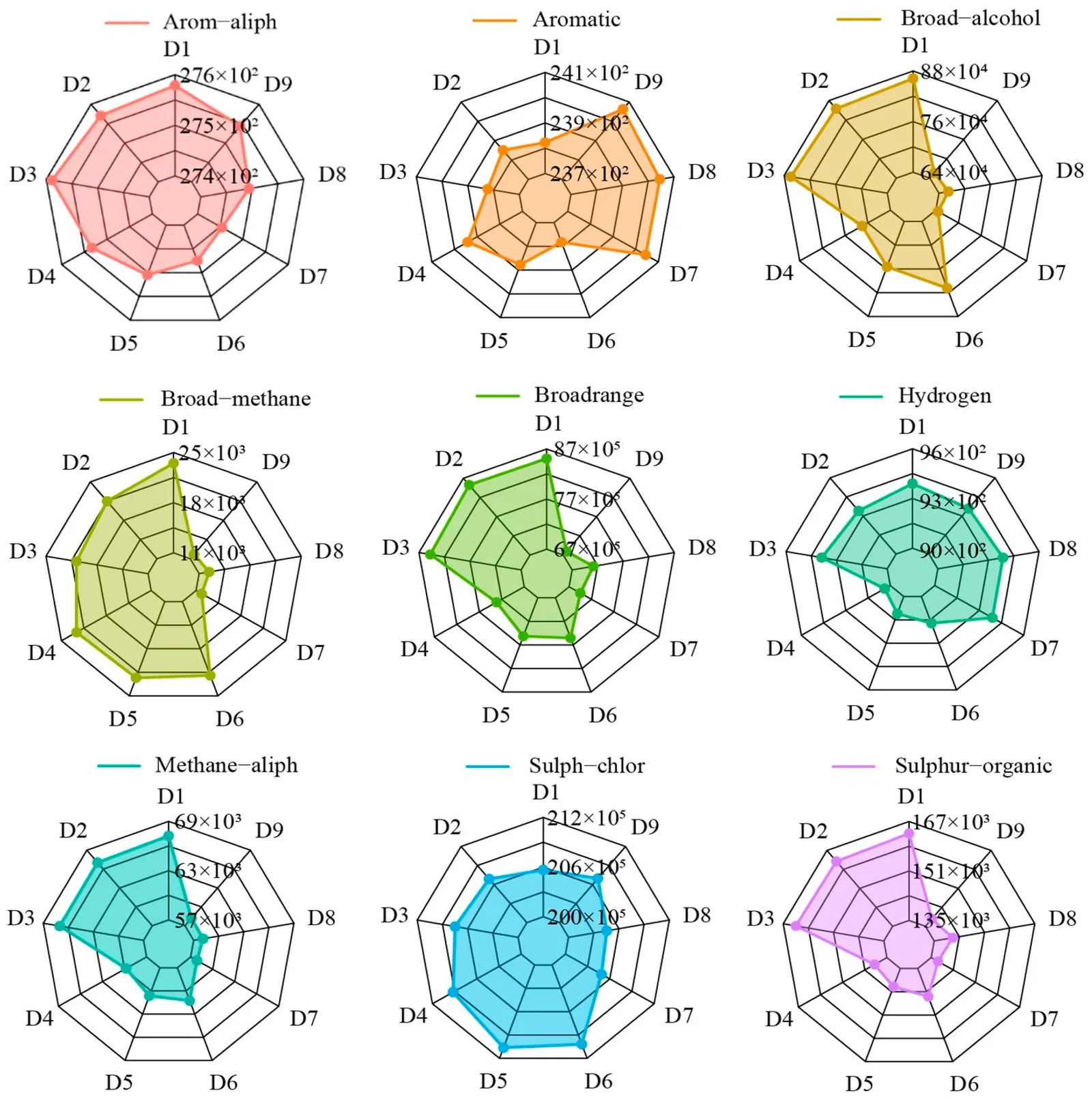
Analysis of volatile components in different Dahongpao tea samples using an electronic nose. D1–D9 denote nine different Dahongpao finished tea products.

In this study, the volatile profiles of nine Dahongpao tea samples (D1–D9) were analyzed using an electronic nose. The sensor response intensities corresponding to nine sensor-specific response categories, namely arom–aliph, aromatic, broad-alcohol, broad-methane, broad range, hydrogen, methane–aliph, sulfur–organic, and sulf–chlor, were obtained. The results of response intensity analysis showed (Figure 3) that for the nine tea samples, the response values ranged from 27,454 to 27,589 (mean 27,527) for arom–aliph, with the highest value observed in D3 and the lowest in D7; for aromatic, the range was 23,783–24,063 (mean 23,917), with D9 being the highest and D6 the lowest; for broad-alcohol, the range was 646,109–864,735 (mean 758,303), with D3 the highest and D7 the lowest; for broad-methane, the range was 11,819–23,532 (mean 18,766), with D1 the highest and D9 the lowest; for broad range, the range was 6,798,313–8,527,592 (mean 7,641,656), with D2 the highest and D9 the lowest; for hydrogen, the range was 9038–9390 (mean 9286), with D1 the highest and D4 the lowest; for methane–aliph, the range was 57,870–67,242 (mean 61,916), with D1 the highest and D7 the lowest; for sulf–chlor, the range was 20,456,834–21,064,120 (mean 20,739,590), with D5 the highest and D8 the lowest; and for sulfur–organic, the range was 137,492–163,151 (mean 147,842), with D1 the highest and D7 the lowest. These results indicate that the nine tea samples selected in this study exhibit distinct advantages in different volatile components, suggesting that the samples are representative to a certain extent.

### 3.2. Analysis of Taste Characteristics of Different Tea Samples

**Figure 4:**
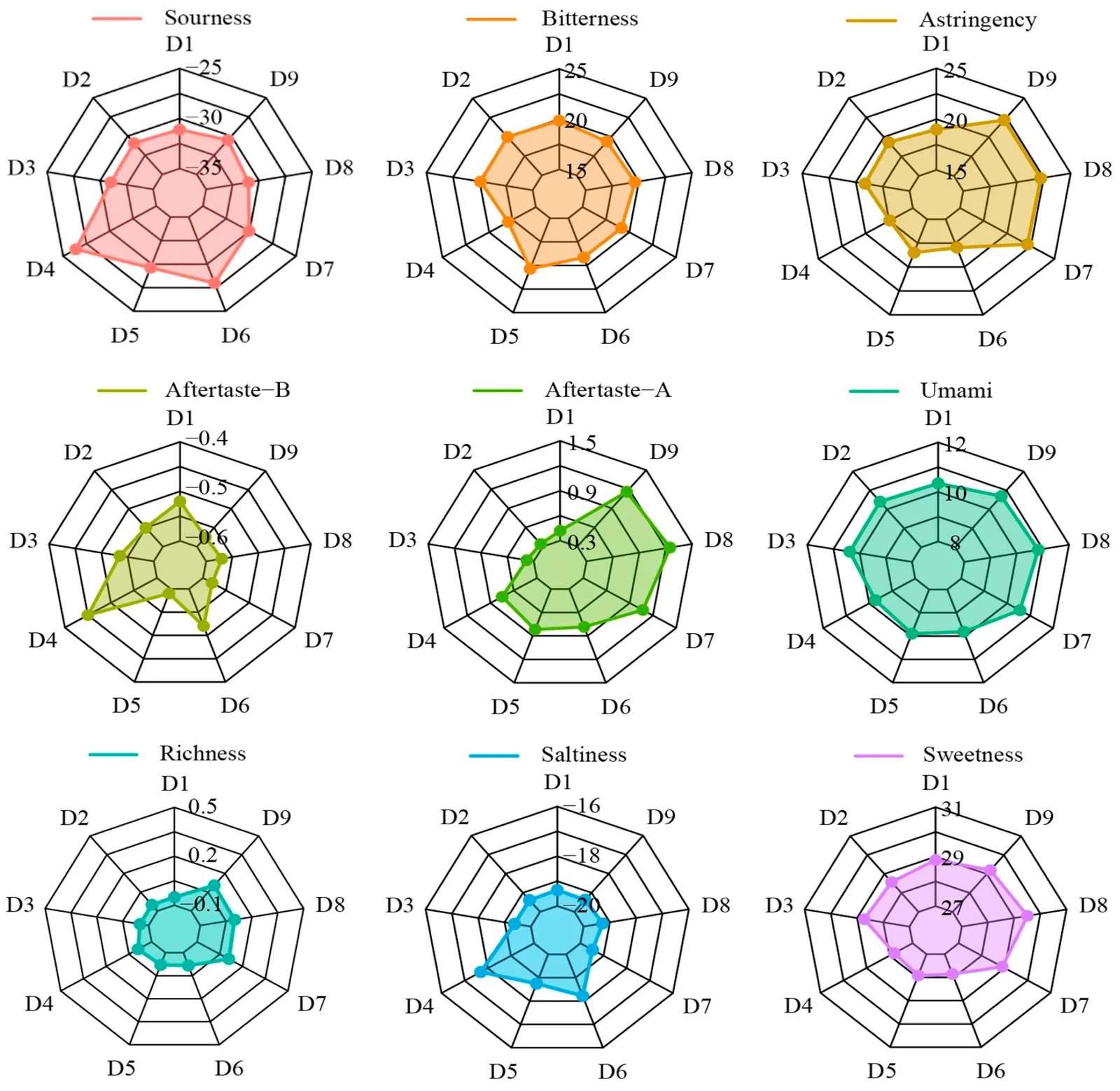
Analysis of taste characteristics in different Dahongpao tea samples using an electronic tongue. D1–D9 denote nine different Dahongpao finished tea products.

In this study, the taste characteristics of nine Dahongpao tea samples (D1–D9) were analyzed using an electronic tongue. The response values for nine taste attributes, namely sourness, bitterness, astringency, aftertaste-B, aftertaste-A, umami, richness, saltiness, and sweetness, were obtained. The results of response intensity analysis showed (Figure 4) that for the nine tea samples, the response values ranged from −31.09 to −26.36 (mean −29.83) for sourness, with the highest value observed in D1 and the lowest in D4; for bitterness, the range was 17.96–20.37 (mean 19.50), with D5 the highest and D4 the lowest; for astringency, the range was 17.44–22.23 (mean 19.77), with D8 the highest and D4 the lowest; for aftertaste-B, the range was −0.59 to −0.45 (mean −0.54), with D5 the highest and D1 the lowest; for aftertaste-A, the range was 0.34–1.25 (mean 0.78), with D8 the highest and D2 the lowest; for umami, the range was 9.74–10.83 (mean 10.30), with D8 the highest and D4 the lowest; for richness, the range was −0.05 to 0.11 (mean 0.01), with D7 the highest and D1 the lowest; for saltiness, the range was −19.47 to −17.69 (mean −18.99), with D7 the highest and D4 the lowest; and for sweetness, the range was 27.8–29.51 (mean 28.60), with D8 the highest and D4 the lowest. These results indicate that the nine tea samples selected in this study exhibit distinct advantages in different taste attributes, suggesting that the samples are representative to a certain extent.

### 3.3. Analysis of Element Contents in Different Tea Samples

In this study, the total contents of 11 elements in tea and their dissolution amounts in tea infusions after five consecutive brews were determined by ICP MS. The results showed (Table 1) that among the nine tea samples, K and Ca had the highest contents, with K ranging from 3944.91 to 5532.93 mg/kg and Ca from 1913.70 to 2909.92 mg/kg. The contents of Mg, Fe, Zn, Mn, and Cu were moderate, ranging from 635.15 to 678.04 mg/kg, 419.32 to 582.17 mg/kg, 355.75 to 558.25 mg/kg, 64.94 to 156.96 mg/kg, and 16.41 to 22.27 mg/kg, respectively. The contents of Na, Se, Cr, and Mo were the lowest, ranging from 594.65 to 741.26 μg/kg, 81.26 to 154.81 μg/kg, 295.38 to 671.54 μg/kg, and 198.54 to 296.51 μg/kg, respectively. During the five consecutive brews, the dissolution amounts of each element in the tea infusions showed distinct stage dependent changes with increasing brew number, and the cumulative dissolution rates after five brews exhibited significant element specific characteristics. Among the elements, Mg had the highest dissolution rate, ranging from 67.24% to 70.94%; Na, Cu, Mn, and Mo all had dissolution rates higher than 25%; Se and Cr exhibited dissolution rates between 15% and 26%; while Fe, Ca, K, and Zn had relatively low dissolution rates, generally below 10%. Further analysis revealed (Table 1) that among the 11 elements, the average total content in tea leaves was highest for K (4532.25 mg/kg), followed by Ca (2415.45 mg/kg) and Mg (652.64 mg/kg). In terms of average dissolution rate, Mg was the highest (69.50%), followed by Na (31.96%) and Cu (31.48%). These results indicated that significant differences existed in both the total contents and dissolution rates of different elements in the tea samples.

### 3.4. Analysis of Changes in Element Dissolution Amounts During Five Consecutive Brews

**Figure 5:**
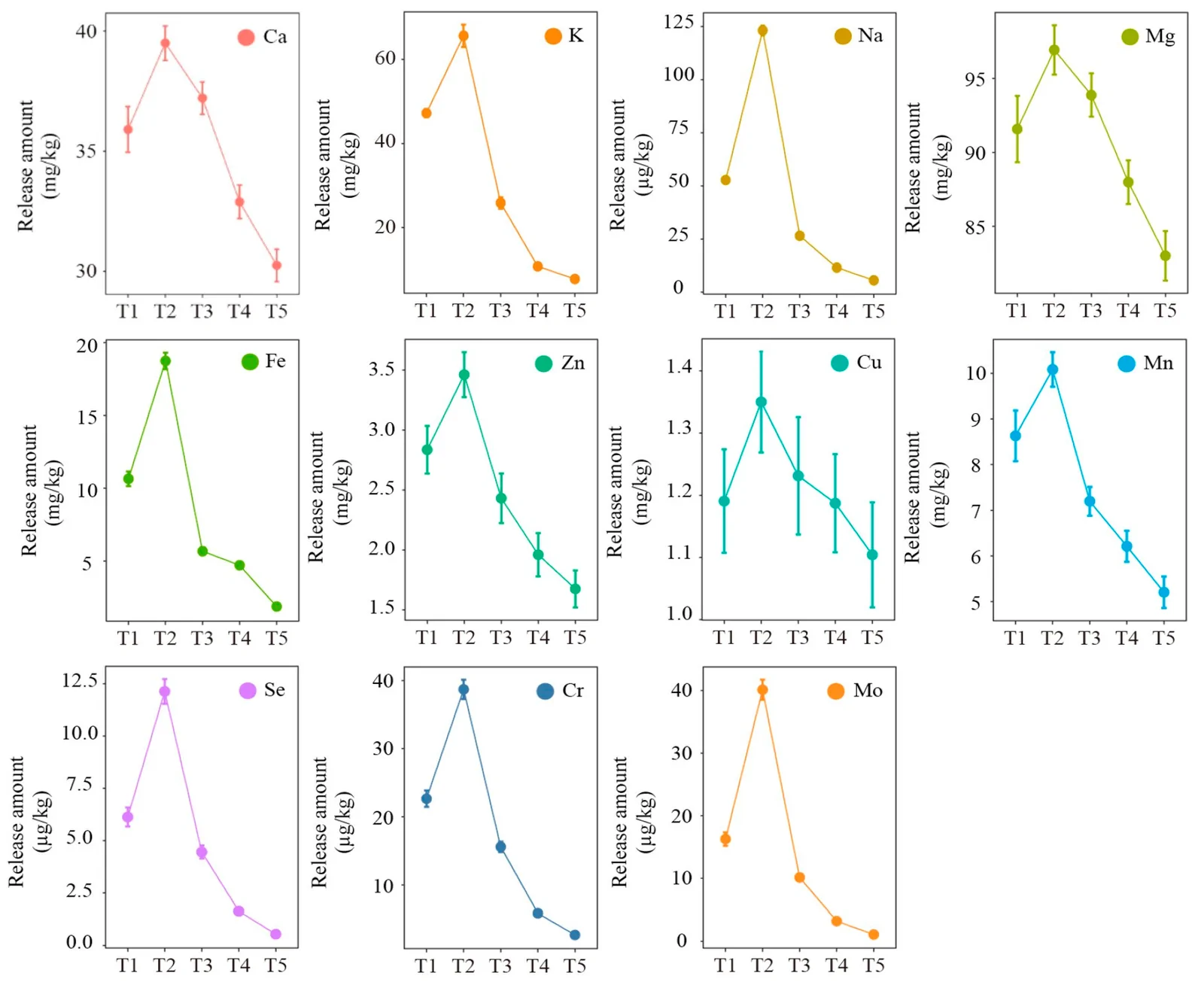
Amounts of elements released from tea leaves during five consecutive brews. T1–T5 denote the tea infusions of the Dahongpao finished tea samples from the first to the fifth brew, respectively.

Based on the above analysis, this study further investigated the changes in dissolution amounts of the 11 elements in tea over five consecutive brews (T1–T5). The results showed (Figure 5) that during the five consecutive brews, the dissolution amounts of all 11 elements exhibited a trend of first increasing and then decreasing rapidly, reaching their maximum at T2. Specifically, from stage T1 to T2, the dissolution amounts of Na (140.74%), Fe (76.32%), Se (96.04%), Cr (74.91%), and Mo (147.36%) showed the most significant increases, whereas those of the other elements increased within the range of 5.85–39.72%. From stage T2 to T5, the dissolution of elements such as K (−89.73%), Na (−95.46%), Fe (−90.27%), Zn (−51.25%), Se (−95.58%), Cr (−93.05%), and Mo (−92.27%) dropped sharply, with reductions exceeding 50%, while those of other elements decreased within the range of 14.35–46.26%. Evidently, the number of infusion cycles exerts a substantial influence on the dissolution of elements from tea, with the first and second infusions contributing the most to element release, and subsequent infusions showing markedly diminished contributions. This pattern indicates that most soluble elements are rapidly released during the first two brewing steps, after which the extractable fraction progressively declines.

**Figure 6:**
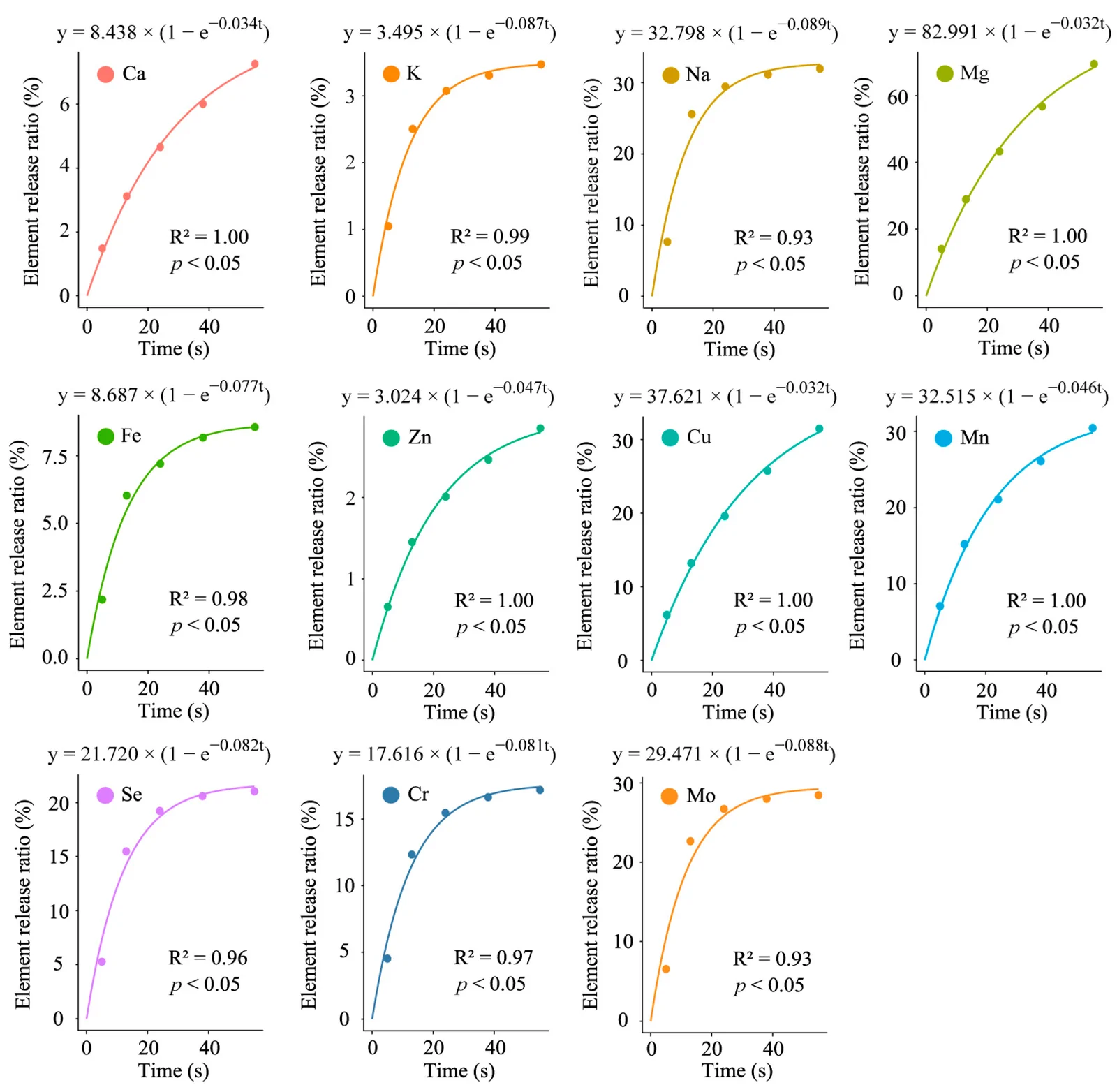
Kinetic modeling of cumulative release rate of different elements in tea versus cumulative brewing time.

Subsequently, kinetic models were used to fit the cumulative element release rate of different elements against cumulative brewing time in order to evaluate whether the dissolution process conformed to kinetic characteristics. The results showed (Figure 6) that the release rate of all 11 elements followed first-order kinetic patterns, with all fits reaching significant levels (R^2^ ranging from 0.93 to 1.00, *p* < 0.05 for all elements). The rate constants (k) varied considerably among elements, with Mg, Ca, and Cu exhibiting the smallest values (approximately 0.032–0.034), indicating a relatively gradual dissolution process with a prolonged plateau phase, whereas K, Na, and Mo displayed larger k values (approximately 0.087–0.089), suggesting rapid initial release followed by a sharp decline. These results indicated that the cumulative release rate of the 11 elements showed a typical first-order kinetic curve pattern, characterized by rapid increase in the early stage, followed by gradual flattening as brewing time extended. This suggested that the dissolution rate was fast initially, then gradually decreased as soluble elements in the tea were depleted, eventually approaching equilibrium, indicating that the dissolution of elements from tea was mainly influenced by brewing time in a regular, time-dependent manner.

**Figure 7:**
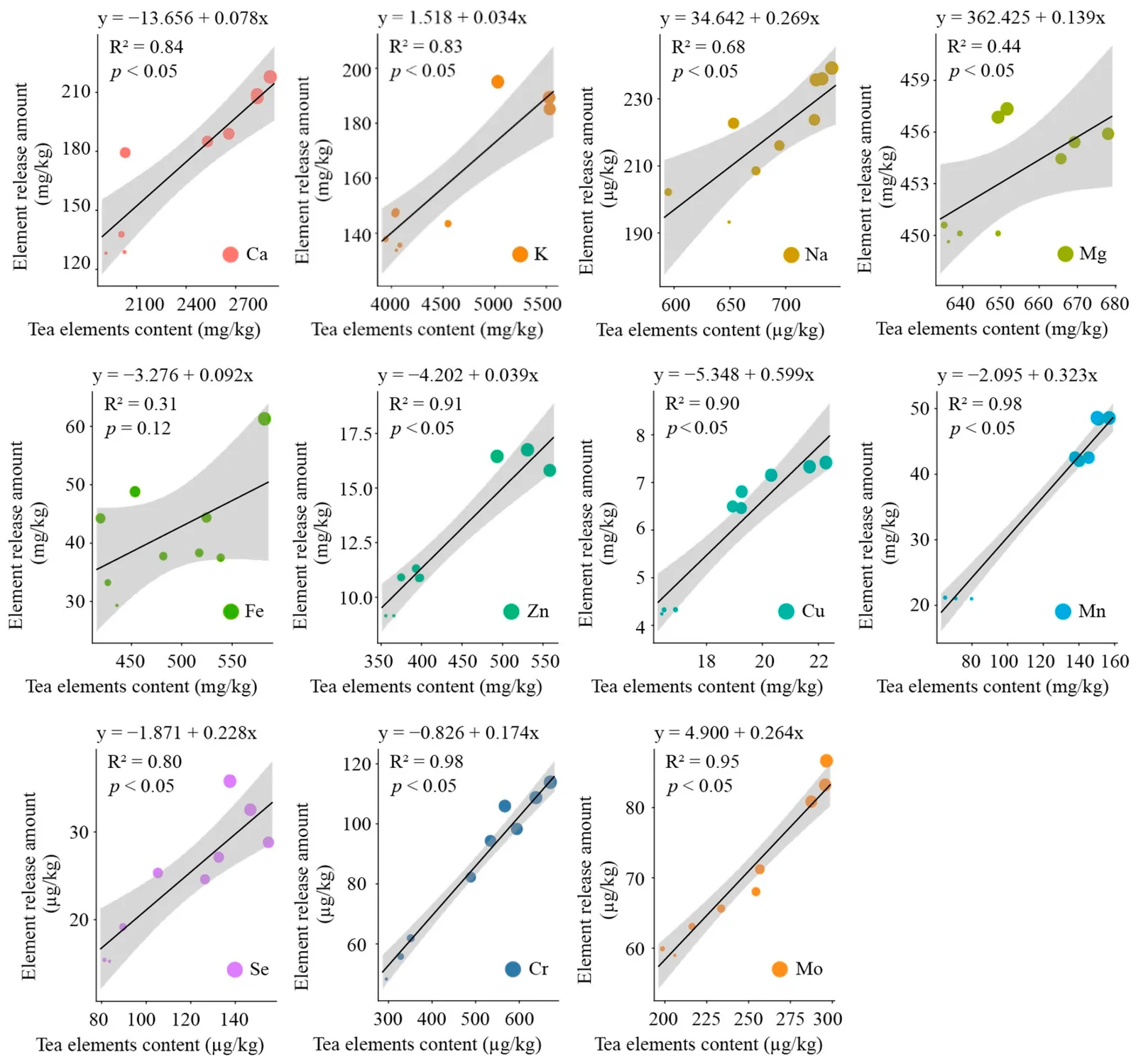
Analysis of the linear regression equation for the total element content and total dissolved amount in tea.

On the basis of the above analysis, a linear regression model was further employed to analyze the relationship between the total element content in tea and the total dissolution amount after five brews. The results showed (Figure 7) that except for Fe (R^2^ = 0.31, *p* = 0.12), the total dissolution amounts of the other 10 elements after five brews were significantly positively correlated with their total contents in tea (R^2^ ranging from 0.44 to 0.98, all *p* < 0.05). Among these, Zn, Cu, Mn, Cr, and Mo showed the strongest correlations (R^2^ > 0.90), indicating that total content is a reliable predictor of dissolution for these elements. These results indicated that, with the exception of Fe, the dissolution amounts of the other elements were significantly influenced by their total contents in tea—that is, higher total contents in tea corresponded to higher dissolution amounts. The exceptional behavior of Fe may be attributed to its strong tendency to form stable insoluble complexes with tea polyphenols, which could render a substantial portion of Fe non-extractable regardless of its total content, thereby decoupling dissolution from total abundance.

### 3.5. Correlation Network Analysis of Total Element Dissolution Rates, Total Contents, and Volatile Profiles in Tea

**Figure 8:**
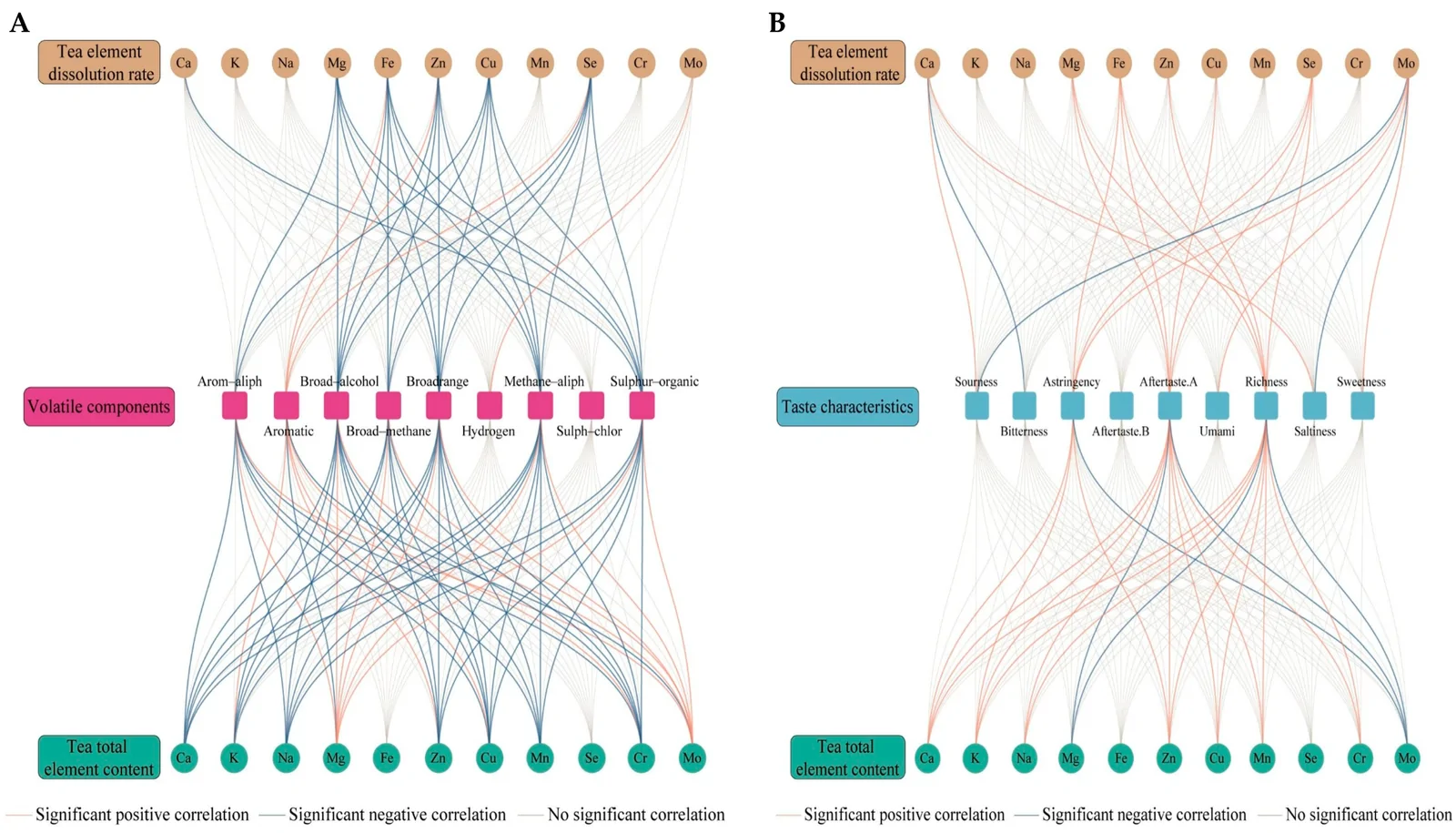
Correlation network of total element dissolution rate, total content, volatile components, and taste characteristics of tea. (**A**) The correlation network between total dissolution rate, total content and volatile components of tea. (**B**) The correlation network between total dissolution rate, total content and taste characteristics of tea.

Based on the above results, correlation network analyses were performed among the total dissolution rates, total contents of the 11 elements in Dahongpao tea, and the nine classes of volatile components. The results showed (Figure 8A) that the total dissolution rates of K, Na, Mn, and Cr were not significantly correlated with any of the nine volatile classes (*p* > 0.05). The total dissolution rates of Mg and Cu were significantly negatively correlated with arom–aliph, broad-alcohol, broad range, methane–aliph, and sulfur–organic; Ca was negatively correlated only with sulfur–organic; Fe and Se were negatively correlated with broad-alcohol, broad-methane, broad range, methane–aliph, and sulfur–organic; Zn was negatively correlated with broad-alcohol, broad-methane, broad range, and methane–aliph; whereas Mo was significantly positively correlated with hydrogen. In addition, the total dissolution rates of Fe, Zn, and Se were significantly positively correlated with aromatic. The correlation network analysis between total element contents and the nine volatile classes indicated (Figure 8A) that the total contents of Ca, K, Na, Mg, Fe, Zn, Cu, Mn, Se, Cr, and Mo were not significantly correlated with sulf–chlor or hydrogen (*p* > 0.05). The total contents of Mg and Mo were significantly positively correlated with sulfur–organic, broad range, broad-methane, broad-alcohol, arom–aliph, and methane–aliph, but negatively correlated with aromatic. The total contents of Ca, Na, Zn, Cu, Mn, and Cr were significantly negatively correlated with sulfur–organic. The total contents of Ca, K, Na, Zn, Cu, Mn, and Cr were negatively correlated with methane–aliph, broad-alcohol, and broad range. The total contents of Ca, K, Zn, Cu, and Cr were significantly positively correlated with aromatic, but negatively correlated with broad-methane. The total contents of Ca, Na, Cu, Mn, and Cr were negatively correlated with arom–aliph. Notably, the above five elements repeatedly appeared in multiple negative correlations, constituting a core group of elements that suppressed multiple volatile classes. Comparing the two types of indicators, the correlation directions of Mg in terms of total dissolution rate and total content were often opposite, suggesting that the soluble fraction and the total reserve of an element may contribute to flavor compounds through fundamentally different mechanisms.

The correlation network analysis between total element dissolution rates, total contents, and taste characteristics revealed (Figure 8B) that the total dissolution rates of K, Na, Mn, and Cr were not significantly correlated with sourness, bitterness, astringency, aftertaste-B, aftertaste-A, umami, richness, saltiness, or sweetness (*p* > 0.05). The total dissolution rate of Ca was significantly positively correlated with saltiness and sourness, but negatively correlated with bitterness. The total dissolution rate of Mg was significantly positively correlated with richness and aftertaste-A. Fe and Se were positively correlated with richness, aftertaste-A, and astringency; Zn was positively correlated with richness; Cu was positively correlated with aftertaste-A; and Mo was negatively correlated with saltiness and sourness, but positively correlated with sweetness, umami, and astringency. The correlation network analysis between total element contents and the nine taste attributes revealed (Figure 8B) that the total contents of Ca, K, Na, Mg, Fe, Zn, Cu, Mn, Se, Cr, and Mo were not significantly correlated with saltiness, sweetness, umami, aftertaste-B, bitterness, or sourness (*p* > 0.05). The total contents of Ca, K, and Zn were significantly positively correlated with astringency, while Mo was negatively correlated with astringency. The total contents of Na, Ca, K, Zn, Cu, Mn, and Cr were significantly positively correlated with aftertaste-A and richness, whereas the total contents of Mg and Mo were significantly negatively correlated with aftertaste-A and richness. Furthermore, Mg showed a positive correlation with aftertaste-A and richness in terms of dissolution rate but a negative correlation in terms of total content, indicating that the same element may exhibit opposite correlation directions between the two types of indicators.

### 3.6. Assessment of Appropriate Daily Tea Consumption for Humans

Based on the average daily intakes of the 11 elements from the daily diet of Chinese residents (Table S1), with reference to the adequate intakes for each element as specified in the 2023 edition of the Chinese Dietary Reference Intakes (Tables S2–S4), and combined with the total dissolution amounts of the 11 elements after five consecutive brews determined in this study, the appropriate daily tea consumption for humans was assessed for each element individually.

For infants aged 0–3 years (Table S5), only children aged 1–3 years could supplement Ca through tea consumption, with an appropriate tea consumption of 851.15 g/d; no appropriate tea consumption was found for the other age groups. K, Na, Mg, Fe, Zn, Cu, Mn, Se, Cr, and Mo were not recommended for supplementation through tea consumption in any age group. Therefore, tea is not a suitable pathway for mineral element supplementation in infants aged 0–3 years.

For adolescents aged 4–17 years (Table S6), the average daily dietary intakes of the elements were relatively stable, whereas the adequate intakes generally increased with age. Ca could be supplemented through tea consumption in the age groups 4–6, 7–8, 9–11, 12–14, and 15–17 years, with appropriate tea consumptions of 1443.46, 2628.08, 3812.70, 3812.70, and 3812.70 g/d, respectively. K could be supplemented only in the 9–11, 12–14, and 15–17 years groups, with tea consumptions of 342.81, 1641.33, and 2939.85 g/d, respectively. Mg could be supplemented only in the 12–14 and 15–17 years groups, with tea consumptions of 121.54 and 143.59 g/d, respectively. Se could be supplemented only in the 9–11, 12–14, and 15–17 years groups, with tea consumptions of 148.46, 803.45, and 803.45 g/d, respectively. Na, Fe, Zn, Cu, Mn, Cr, and Mo were not suitable for supplementation through tea consumption in any age group. Adolescents could supplement small amounts of Ca, K, Mg, and Se through tea, and the required tea consumption increased with age; however, tea serves only as a supplementary approach and cannot replace daily dietary intake.

For pregnant and lactating women (Table S7), Ca could be supplemented in all stages, with an appropriate tea consumption of 2628.08 g/d. For K, the appropriate tea consumption was 2939.85 g/d in the first, second, and third trimesters, and 5536.89 g/d during lactation. For Mg, the appropriate tea consumption was 231.82 g/d in all trimesters and 143.59 g/d during lactation. Fe could be supplemented only in the second trimester, third trimester, and lactation, with tea consumptions of 100.17, 200.34, and 75.13 g/d, respectively. Zn could be supplemented in all trimesters (17.03 g/d) and during lactation (229.87 g/d). Cu could be supplemented only during lactation, with a tea consumption of 52.14 g/d. Se could be supplemented in all trimesters (1021.77 g/d) and during lactation (1589.43 g/d). Na, Mn, Cr, and Mo were not suitable for supplementation through tea consumption.

For males aged 18 years and above (Table S8), the adequate intake of Ca was 800 mg/d for all age groups, corresponding to a tea consumption of 2628.08 g/d. For K, the adequate intake was 2000 mg/d, corresponding to 2939.85 g/d of tea. For Mg, the adequate intake decreased slightly with age, from 330 mg/d in the 18–29 years group to 300 mg/d in the >75 years group, with tea consumption correspondingly decreasing from 143.59 to 77.42 g/d. For Zn, the adequate intake was 12.5 mg/d for the 18–29 years group (tea consumption 187.30 g/d) and 12 mg/d for other age groups (tea consumption 144.73 g/d). For Se, the adequate intake was 0.060 mg/d for all age groups, with a tea consumption of 803.45 g/d. Na, Fe, Cu, Mn, Cr, and Mo were not suitable for supplementation through tea consumption in any age group.

For females aged 18 years and above, including elderly women (Table S9), the adequate intake of Ca was 800 mg/d for all age groups, all of which could be supplemented with a tea consumption of 2628.08 g/d. For K, the adequate intake was 2000 mg/d, with a tea consumption of 2939.85 g/d. For Se, the adequate intake was 0.06 mg/d, with a tea consumption of 803.45 g/d. For Mg, the adequate intake decreased from 330 mg/d in the 18–29 years group to 300 mg/d in the >75 years group, with tea consumption correspondingly decreasing from 143.59 to 77.42 g/d. Fe, Zn, Cu, Mn, Cr, Mo, and Na were not suitable for supplementation through tea consumption.

## 4. Discussion

In this study, the contents of 11 mineral elements in nine Dahongpao tea samples and their dissolution patterns during five consecutive brews were systematically analyzed. In combination with electronic nose and electronic tongue techniques, the interactions among element dissolution rates, element contents, volatile components, and taste characteristics of tea were also investigated. Furthermore, based on the Dietary Reference Intakes, the appropriate daily tea consumption for different population groups was assessed. The results provide data support for understanding the release behavior of mineral elements in Dahongpao tea and their potential contributions to tea infusion flavor, and also offer a reference for rational tea consumption.

### 4.1. Elemental Content Characteristics of Tea and Dissolution Patterns During Brewing

In this study, it was found that K and Ca had the highest contents in Dahongpao tea, followed by Mg, Fe, Zn, and Na, while the contents of trace elements such as Se, Cr, and Mo were relatively low. This distribution pattern is similar to that of most previously reported tea elemental compositions [25,26], reflecting the enrichment characteristics of tea plants for K and Ca and their important roles in physiological metabolism. During the five consecutive brews, the dissolution amounts of most elements reached their peak after the first to second infusion and then gradually decreased. This phenomenon is similar to the dissolution patterns of elements observed in different types of tea after brewing, indicating that although tea types and appearances may differ, there is a certain commonality in the element dissolution patterns [27,28]. Furthermore, this study found that Mg had the highest dissolution rate (67.24–70.94%), significantly higher than that of macronutrients such as K and Ca; in contrast, the dissolution rates of Fe, Ca, K, and Zn were below 10%. The high dissolution rate of Mg in tea infusion is mainly attributed to its structural localization as the core element of chlorophyll and its ionic properties; Mg is the central ion of the chlorophyll porphyrin ring and is tightly bound to chlorophyll molecules in fresh tea leaves [29]. During tea processing, especially fermentation, chlorophyll undergoes degradation under enzymatic oxidation and hydrothermal conditions, releasing Mg from the porphyrin ring and converting it into free Mg^2+^ or soluble small-molecule organic magnesium salts that enter the cell sap [30], thereby making it easier to dissolve during the brewing process. The low dissolution rates of Fe, Ca, K, and Zn during brewing may be mainly attributed to the following reasons: Fe readily forms stable colored complexes with polyphenols, making it difficult to release; Ca is immobilized due to cross-linking with cell wall pectin and complexation with tea polyphenols; and Zn is subject to the dual effects of polyphenol complexation and cell wall structure [31,32]. Furthermore, the dissolution rate of K observed in this study (below 10%) is largely a reflection of its exceptionally high total content in the tea matrix rather than an indication of poor extractability. The absolute amount of K released was still markedly higher than that of most other elements. In addition, the compact appearance of Dahongpao tea resulting from its unique roasting process may physically limit water penetration and ion diffusion during the short brewing times applied in this study, thereby contributing to the relatively low percentage extraction. Unlike Mg, which is released from chlorophyll degradation products, a considerable portion of K may exist in less readily extractable forms, such as those associated with cell wall components via electrostatic interactions or occluded within dense cellular structures formed during processing [33]. Collectively, these results indicate that the dissolution rates of different elements in tea leaves vary significantly, and the difference between the high dissolution rate of Mg and the low dissolution rates of Fe, Ca, K, and Zn originates from the different occurrence forms of these elements and their distinct binding modes with cell walls and polyphenols. Moreover, from the perspective of nutritional supplementation, elements with high dissolution rates may be more readily absorbed by the human body through tea consumption, whereas elements with low dissolution rates are difficult to effectively obtain via brewing.

### 4.2. Kinetic Characteristics of Element Dissolution During Tea Brewing

The kinetic fitting results of this study showed that the cumulative dissolution amounts of all 11 elements conformed to the first order kinetic model. This result indicates that the dissolution process of elements from tea is controlled by diffusion driven by concentration gradients, with a relatively rapid initial dissolution rate that gradually approaches equilibrium as the soluble components decrease with increasing brew number [34]. Wang et al. [35] determined the contents of Na, Fe, Zn, Cu, Mn, and the hazardous elements Bi, Cd, Pb, and Hg in tea and tea infusions from Honghe Prefecture, Yunnan Province, and similarly found that element contents in tea infusions increased with prolonged steeping time and exhibited a trend of first increasing and then decreasing with increasing brew number. This suggests that the release behavior of mineral elements is consistent with the overall dissolution pattern of soluble substances in the tea matrix. Furthermore, this study found that the first order rate constants (k values) varied significantly among the different elements, ranging from 0.032 to 0.089. Specifically, Mg and Ca had relatively small k values (0.032 and 0.034), whereas K, Na, and Mo had larger k values (0.087 and 0.089). A smaller k value indicates a relatively gentler dissolution process with a delayed peak or a longer plateau phase, whereas a larger k value suggests that the element is released rapidly in the initial stage and then declines quickly [36]. This difference may be attributed to the distinct localization and binding status of each element within the tea leaf tissues.

### 4.3. Relationship Between Total Element Content in Tea and Dissolution Amount

The linear regression analysis results of this study showed that, except for Fe, the total dissolution amounts of the other 10 elements were significantly positively correlated with their total contents in tea. This result indicates that, for most mineral elements, the total content in tea is one of the major factors determining their dissolution amount [37]. Dong et al. [38] investigated the composition of trace elements in tea and the relationships between dissolution rate and brewing method, brewing time, and brewing temperature, and concluded that the dissolution rate of elements decreases with increasing brew number, with significant differences among elements, and that the total content of an element in tea is generally positively correlated with its dissolution amount, although this correlation is influenced by chemical speciation. The exceptional behavior of Fe may be closely related to its chemical form in tea, as Fe readily forms stable insoluble complexes with tea polyphenols, causing its dissolution behavior to deviate from the regulation by total content [39,40]. Collectively, these findings suggest that selecting tea raw materials with high elemental contents can, to a certain extent, increase the content of mineral elements in tea infusions; however, for elements such as Fe that are significantly influenced by chemical speciation, simply increasing the total content may not effectively enhance dissolution, and the effects of processing methods and brewing conditions should also be taken into consideration.

### 4.4. Interactions Between Element Contents in Tea and Tea Flavor

In this study, correlation network analysis was employed to investigate the potential associations between total element contents and total dissolution rates with the volatile components and taste characteristics of tea, and several “element clusters” with suggestive correlational patterns were detected. The total dissolution rates of Fe, Zn, and Se exhibited significantly positive correlations with aromatic, while those of Mg and Cu showed significantly negative correlations with sulfur–organic and methane–aliph. Furthermore, the total dissolution rate of Ca was significantly positively correlated with saltiness and sourness, but negatively correlated with bitterness; the total dissolution rates of Mg and Fe were significantly positively correlated with richness and aftertaste-A. Bai et al. [41] found that mineral content was a major factor affecting volatile compounds in green tea infusions, with Ca^2+^ and Fe^3+^ significantly influencing the contents of alcohols and aldehydes among volatile compounds. Polat et al. [42] evaluated the mineral contents and sensory parameters of tea infusions under different brewing times and found that turbidity of tea infusion was positively correlated with Zn, Ca, and Mn, while brightness was negatively correlated with Al; steeping time was positively correlated with color and bitterness, but negatively correlated with brightness, clarity, astringency, aroma, and overall acceptability. Collectively, these findings suggest that the correlations between elements and tea flavor are not simply determined by their total contents, but rather the actual soluble proportion may also show associations with both volatile components and taste characteristics. However, it is important to emphasize that the statistically significant correlations observed in this network analysis are exploratory and do not demonstrate a direct causal or mechanistic role of mineral ions in taste or aroma perception.

### 4.5. Population-Based Evaluation of Appropriate Tea Consumption

The assessment of appropriate daily tea consumption based on the Chinese Dietary Reference Intakes for residents showed that, with the exception of a few elements such as Ca, K, Mg, and Se, the vast majority of mineral elements could not be effectively supplemented through normal tea consumption. Even for Ca, the required tea consumption for infants, adolescents, and adults ranged from hundreds to thousands of grams per day, far exceeding the practically feasible tea consumption (typically 3–10 g/d) [43]. Thus, tea is not an ideal vehicle for supplementing macro- or micro-elements such as Ca, Fe, or Zn. For Mg and Se, the appropriate tea consumption for certain age groups (e.g., 15–17 years and adult males) decreased to 77–144 g/d; although this amount still exceeds daily tea consumption in practice, it suggests that under long-term, high-frequency, and large-volume tea drinking habits, tea may contribute as an auxiliary source for Mg and Se intake. Pregnant and lactating women have increased requirements for Fe, Zn, and Cu; the estimated appropriate tea consumption based on a single-element perspective in this study remained relatively high and could not be met by tea alone, and priority should be given to dietary supplementation. Collectively, beyond the 11 elements measured in this study, tea contains numerous other mineral elements and trace components that were not included in this assessment, and factors such as element speciation, bioaccessibility, and interactions with food matrices were also not considered; the population assessment relied on literature-reported average dietary intake values and did not account for individual variations or the fluctuations in element contents of tea from different origins. The role of mineral elements in tea for nutritional supplementation is limited, and its benefits should not be overstated. Consumers should choose their tea consumption amount reasonably according to their age and physiological status, and special attention should be paid to infants, children, and adolescents to avoid replacing regular meals with tea.

## 5. Conclusions

In this study, the contents of 11 mineral elements in Dahongpao tea, their dissolution patterns during five consecutive brews, the kinetic characteristics of dissolution, and their interactions with volatile components and taste characteristics were systematically investigated. In addition, the appropriate daily tea consumption for different population groups was assessed. The results showed that K and Ca had the highest contents in Dahongpao tea, Mg exhibited the highest dissolution rate, while Fe, Ca, K, and Zn showed the lowest dissolution rates. The cumulative dissolution amounts of all elements conformed to the first-order kinetic model, and with the exception of Fe, the total dissolution amounts were significantly positively correlated with the total element contents in tea leaves. Correlation network analysis revealed that elements such as Mg and Mo were broadly associated with multiple classes of volatile components, while Ca, Mg, and Fe exhibited potential modulatory effects on taste attributes including richness and aftertaste-A. The population-based assessment indicated that only Ca, K, Mg, and Se could be supplemented to a minor extent through tea consumption; however, the required tea consumption far exceeded the amounts typically consumed in daily life, and tea is not recommended as a major dietary source for the supplementation of most mineral elements. This study provides an important foundation for understanding the release behavior of mineral elements in Dahongpao tea and their potential contributions to tea infusion flavor. Nevertheless, the correlation network analysis in this study represents only a preliminary exploration of the interactions and lacks experimental validation of direct causal relationships between elements and flavor compounds. Future studies should incorporate in vitro digestion models, sensory addition experiments, and larger scale dietary surveys to further verify the bioaccessibility of mineral elements in tea and their actual mechanisms contributing to flavor.

## Figures and Tables

**Table 1:** ICP-MS determination results of different elements in tea and tea infusions.

Element	The Distribution Range of the Total Amount of Elements	The Average of the Total Amount of Elements	After the First to Fifth Infusions, the Distribution Range of the Element Release Amounts in the Tea Infusion					Distribution Range of Total Element Dissolution Rate (%)	Average of Total Element Dissolution Rate (%)
			T1	T2	T3	T4	T5		
Ca (mg/kg)	1913.70~2909.92	2415.45 ± 415.22	26.31~45.96	28.95~48.96	28.65~43.82	22.22~41.87	22.06~37.41	6.35~8.83	7.26 ± 0.69
K (mg/kg)	3944.91~5532.93	4532.25 ± 662.19	40.49~53.19	55.09~86.09	20.61~33.21	4.80~17.42	4.58~14.57	3.16~3.88	3.47 ± 0.22
Na (μg/kg)	594.65~741.26	687.92 ± 49.31	34.67~67.76	114.60~132.86	21.43~29.78	9.67~13.16	4.10~6.60	29.76~34.09	31.96 ± 1.45
Mg(mg/kg)	635.15~678.04	652.64 ± 15.28	90.51~93.40	94.84~98.21	90.51~95.80	85.69~89.54	82.32~83.76	67.24~70.94	69.50 ± 1.34
Fe (mg/kg)	419.32~582.17	486.61 ± 57.08	8.58~13.68	13.85~26.37	3.44~9.26	2.70~8.85	0.74~3.14	6.73~10.76	8.56 ± 1.62
Zn (mg/kg)	355.75~558.25	429.64 ± 76.45	1.88~3.74	2.48~4.82	1.88~3.46	1.52~2.62	1.16~2.30	2.50~3.34	2.85 ± 0.26
Cu (mg/kg)	16.41~22.27	19.05 ± 2.16	0.85~1.53	0.94~1.60	0.83~1.47	0.78~1.43	0.75~1.39	25.63~35.35	31.48 ± 4.23
Mn(mg/kg)	64.94~156.96	121.91 ± 38.16	4.82~10.90	5.29~13.39	4.07~9.78	3.42~8.86	3.30~6.65	26.34~32.63	30.46 ± 1.96
Se (μg/kg)	81.26~154.81	117.51 ± 28.17	4.29~8.46	7.23~18.83	2.42~6.22	0.88~2.19	0.26~0.78	18.26~26.03	21.05 ± 2.65
Cr (μg/kg)	295.38~671.54	496.65 ± 139.97	11.73~30.12	22.60~51.95	8.80~21.69	3.45~8.46	1.41~4.23	16.35~18.68	17.18 ± 0.71
Mo (μg/kg)	198.54~296.51	249.44 ± 38.26	13.32~20.23	32.85~49.84	8.04~12.48	2.53~4.57	0.90~1.24	26.75~30.18	28.45 ± 0.99

Note: T1–T5 denote the tea infusions of the Dahongpao finished tea samples from the first to the fifth brew, respectively.

## Data Availability

The original contributions presented in this study are included in the article/Supplementary Materials. Further inquiries can be directed to the corresponding authors.

## Supplementary Materials

The following supporting information can be downloaded at https://www.mdpi.com/article/10.3390/foods15173119/s1, Table S1. Average daily dietary intakes of different elements for Chinese residents. Table S2. Recommended intake levels of different elements for infants aged 0–3 years and adolescents aged 4–17 years. Table S3. Recommended intake levels of different elements for pregnant women. Table S4. Recommended intake levels of different elements for males and females aged 18 years and above. Table S5. Appropriate tea consumption to supplement elements for infants aged 0–3 years based on average daily dietary intakes. Table S6. Appropriate tea consumption to supplement elements for adolescents aged 4–17 years based on average daily dietary intakes. Table S7. Appropriate tea consumption to supplement elements for pregnant women based on average daily dietary intakes. Table S8. Appropriate tea consumption to supplement elements for males aged 18 years and above based on average daily dietary intakes. Table S9. Appropriate tea consumption to supplement elements for females aged 18 years and above based on average daily dietary intakes.
