# Supplementary material for: Dissolution Behavior of Mineral Elements in Dahongpao Tea, Its Association with Flavor Quality, and Potential Contribution of Infusions to Dietary Reference Intakes
Source: Foods. 2026 Sep 2;15(17):3119. doi: 10.3390/foods15173119 (PMC13565726; doi:10.3390/foods15173119)
Supplement: Supplementary file 1 [file foods-15-03119-s001.zip › foods-4496147-supplementary.pdf]

# Supplementary Materials

Table S1. Average daily dietary intakes of different elements for Chinese residents.

Table S2. Recommended intake levels of different elements for infants aged 0-3 years and adolescents aged 4-17 years.

Table S3. Recommended intake levels of different elements for pregnant women.

Table S4. Recommended intake levels of different elements for males and females aged 18 years and above.

Table S5. Appropriate tea consumption to supplement elements for infants aged 0-3 years based on average daily dietary intakes.

Table S6. Appropriate tea consumption to supplement elements for adolescents aged 4-17 years based on average daily dietary intakes.

Table S7. Appropriate tea consumption to supplement elements for pregnant women based on average daily dietary intakes.

Table S8. Appropriate tea consumption to supplement elements for males aged 18 years and above based on average daily dietary intakes.

Table S9. Appropriate tea consumption to supplement elements for females aged 18 years and above based on average daily dietary intakes.

**Table S1. Average daily dietary intakes of different elements for Chinese residents.**

| **Element** | **Ca (mg/d)** | **K (mg/d)** | **Na (mg/d)** | **Mg (mg/d)** | **Fe (mg/d)** | **Zn (mg/d)** |
|---|---|---|---|---|---|---|
| Recommended intake | 356.3 | 1547.2 | 6046.0 | 264.9 | 21.0 | 10.3 |
| **Element** | **Cu (mg/d)** | **Mn (mg/d)** | **Se (μg/d)** | **Cr (μg/d)** | **Mo (μg/d)** | |
| Recommended intake | 1.2 | 4.8 | 41.6 | 139.1 | 161.0 | |

**Table S2. Recommended intake levels of different elements for infants aged 0-3 years and adolescents aged 4-17 years.**

| Element | Infants aged 0-3 years | | | Adolescents aged 4-17 years | | | | |
|---|---|---|---|---|---|---|---|---|
| | 0-0.5 | 0.5-1 | 1-3 | 4-6 | 7-8 | 9-11 | 12-14 | 15-17 |
| Ca (mg/d) | 200 | 350 | 500 | 600 | 800 | 1000 | 1000 | 1000 |
| K (mg/d) | 400 | 600 | 900 | 1100 | 1300 | 1600 | 1800 | 2000 |
| Na (mg/d) | 80 | 180 | 500 | 800 | 900 | 1100 | 1400 | 1600 |
| Mg (mg/d) | 20 | 65 | 140 | 160 | 200 | 250 | 320 | 330 |
| Fe (mg/d) | 0.3 | 0.3 | 10 | 10 | 12 | 16 | 16 | 16 |
| Zn (mg/d) | 1.5 | 3.2 | 4 | 5.5 | 7 | 7 | 7.5 | 8 |
| Cu (mg/d) | 0.3 | 0.3 | 0.3 | 0.4 | 0.5 | 0.6 | 0.7 | 0.8 |
| Mn (mg/d) | 0.01 | 0.7 | 1.5 | 2 | 2.5 | 3 | 4 | 4 |
| Se (μg/d) | 15 | 20 | 25 | 30 | 40 | 45 | 60 | 60 |
| Cr (μg/d) | 0.2 | 5 | 15 | 15 | 20 | 25 | 30 | 30 |
| Mo (μg/d) | 3 | 6 | 10 | 12 | 15 | 20 | 25 | 25 |

**Table S3. Recommended intake levels of different elements for pregnant women.**

| Element | Early pregnancy | Mid-pregnancy | Late pregnancy | Lactation period |
|---|---|---|---|---|
| Ca (mg/d) | 800 | 800 | 800 | 800 |
| K (mg/d) | 2000 | 2000 | 2000 | 2400 |
| Na (mg/d) | 1500 | 1500 | 1500 | 1500 |
| Mg (mg/d) | 370 | 370 | 370 | 330 |
| Fe (mg/d) | 18 | 25 | 29 | 24 |
| Zn (mg/d) | 10.5 | 10.5 | 10.5 | 13 |
| Cu (mg/d) | 0.91 | 0.9 | 0.9 | 1.5 |
| Mn (mg/d) | 4 | 4 | 4 | 4.2 |
| Se (μg/d) | 65 | 65 | 65 | 78 |
| Cr (μg/d) | 30 | 33 | 35 | 35 |
| Mo (μg/d) | 25 | 25 | 25 | 30 |

**Table S4. Recommended intake levels of different elements for males and females aged 18 years and above.**

| Element | Males aged 18 to 64 | | | Males aged over 65 | | Females aged 18 to 64 | | | Females aged over 65 | |
|---|---|---|---|---|---|---|---|---|---|---|
| | 18-29 | 30-49 | 50-64 | 65-74 | Over 75 | 18-29 | 30-49 | 50-64 | 65-74 | Over 75 |
| Ca (mg/d) | 800 | 800 | 800 | 800 | 800 | 800 | 800 | 800 | 800 | 800 |
| K (mg/d) | 2000 | 2000 | 2000 | 2000 | 2000 | 2000 | 2000 | 2000 | 2000 | 2000 |
| Na (mg/d) | 1500 | 1500 | 1500 | 1400 | 1400 | 1500 | 1500 | 1500 | 1400 | 1400 |
| Mg (mg/d) | 330 | 320 | 320 | 310 | 300 | 330 | 320 | 320 | 310 | 300 |
| Fe (mg/d) | 12 | 12 | 12 | 12 | 12 | 18 | 18 | 18 | 10 | 10 |
| Zn (mg/d) | 12.5 | 12 | 12 | 12 | 12 | 8.5 | 8.5 | 8.5 | 8.5 | 8.5 |
| Cu (mg/d) | 0.8 | 0.8 | 0.8 | 0.8 | 0.7 | 0.8 | 0.8 | 0.8 | 0.8 | 0.7 |
| Mn (mg/d) | 4.5 | 4.5 | 4.5 | 4.5 | 4.5 | 4 | 4 | 4 | 4 | 4 |
| Se (μg/d) | 60 | 60 | 60 | 60 | 60 | 60 | 60 | 60 | 60 | 60 |
| Cr (μg/d) | 35 | 35 | 30 | 30 | 30 | 30 | 30 | 25 | 25 | 25 |
| Mo (μg/d) | 25 | 25 | 25 | 25 | 25 | 25 | 25 | 25 | 25 | 25 |

**Table S5. Appropriate tea consumption to supplement elements for infants aged 0-3 years based on average daily dietary intakes.**

| Element | Average daily normal intake（mg/d） | 0-0.5 years | | 0.5-1 years | | 1-3 years | |
|---|---|---|---|---|---|---|---|
| | | Recommended intake（mg/d） | Appropriate tea consumption（g/d） | Recommended intake（mg/d） | Appropriate tea consumption（g/d） | Recommended intake（mg/d） | Appropriate tea consumption（g/d） |
| Ca | 356.3 | 200 | —— | 350 | —— | 500 | 851.15±188.53 |
| K | 1547.2 | 400 | —— | 600 | —— | 900 | —— |
| Na | 6046.0 | 80 | —— | 180 | —— | 500 | —— |
| Mg | 264.9 | 20 | —— | 65 | —— | 140 | —— |
| Fe | 21.0 | 0.3 | —— | 0.3 | —— | 10 | —— |
| Zn | 10.3 | 1.5 | —— | 3.2 | —— | 4 | —— |
| Cu | 1.2 | 0.3 | —— | 0.3 | —— | 0.3 | —— |
| Mn | 4.8 | 0.01 | —— | 0.7 | —— | 1.5 | —— |
| Se | 0.0416 | 0.015 | —— | 0.020 | —— | 0.025 | —— |
| Cr | 0.1391 | 0.0002 | —— | 0.005 | —— | 0.015 | —— |
| Mo | 0.1610 | 0.003 | —— | 0.006 | —— | 0.010 | —— |

Note: —— indicates that it is not advisable to obtain these elements through tea consumption.

**Table S6. Appropriate tea consumption to supplement elements for adolescents aged 4-17 years based on average daily dietary intakes.**

| Element | Average daily normal intake （mg/d） | 4-6 years | | 7-8 years | | 9-11 years | | 12-14 years | | 15-17 years | |
|---|---|---|---|---|---|---|---|---|---|---|---|
| | | Recommended intake （mg/d） | Appropriate tea consumption （g/d） | Recommended intake （mg/d） | Appropriate tea consumption （g/d） | Recommended intake （mg/d） | Appropriate tea consumption （g/d） | Recommended intake （mg/d） | Appropriate tea consumption （g/d） | Recommended intake （mg/d） | Appropriate tea consumption （g/d） |
| Ca | 356.3 | 600 | 1443.46±319.72 | 800 | 2628.08±582.11 | 1000 | 3812.70±844.49 | 1000 | 3812.70±844.49 | 1000 | 3812.70±844.49 |
| K | 1547.2 | 1100 | —— | 1300 | —— | 1600 | 342.81±50.18 | 1800 | 1641.33±240.26 | 2000 | 2939.85±430.33 |
| Na | 6046.0 | 800 | —— | 900 | —— | 1100 | —— | 1400 | —— | 1600 | —— |
| Mg | 264.9 | 160 | —— | 200 | —— | 250 | —— | 320 | 121.54±0.86 | 330 | 143.59±1.02 |
| Fe | 21.0 | 10 | —— | 12 | —— | 16 | —— | 16 | —— | 16 | —— |
| Zn | 10.3 | 5.5 | —— | 7 | —— | 7 | —— | 7.5 | —— | 8 | —— |
| Cu | 1.2 | 0.4 | —— | 0.5 | —— | 0.6 | —— | 0.7 | —— | 0.8 | —— |
| Mn | 4.8 | 2 | —— | 2.5 | —— | 3 | —— | 4 | —— | 4 | —— |
| Se | 0.0416 | 0.030 | —— | 0.040 | —— | 0.045 | 148.46±47.65 | 0.060 | 803.45±257.88 | 0.060 | 803.45±257.88 |
| Cr | 0.1391 | 0.015 | —— | 0.020 | —— | 0.025 | —— | 0.030 | —— | 0.030 | —— |
| Mo | 0.1610 | 0.012 | —— | 0.015 | —— | 0.020 | —— | 0.025 | —— | 0.025 | —— |

Note: —— indicates that it is not advisable to obtain these elements through tea consumption.

**Table S7. Appropriate tea consumption to supplement elements for pregnant women based on average daily dietary intakes.**

| Element | Average daily normal intake (mg/d) | Early pregnancy | | Mid-pregnancy | | Late pregnancy | | Lactation period | |
|---|---|---|---|---|---|---|---|---|---|
| | | Recommended intake (mg/d) | Appropriate tea consumption (g/d) | Recommended intake (mg/d) | Appropriate tea consumption (g/d) | Recommended intake (mg/d) | Appropriate tea consumption (g/d) | Recommended intake (mg/d) | Appropriate tea consumption (g/d) |
| Ca | 356.3 | 800 | 2628.08±582.11 | 800 | 2628.08±582.11 | 800 | 2628.08±582.11 | 800 | 2628.08±582.11 |
| K | 1547.2 | 2000 | 2939.85±430.33 | 2000 | 2939.85±430.33 | 2000 | 2939.85±430.33 | 2400 | 5536.89±810.49 |
| Na | 6046.0 | 1500 | —— | 1500 | —— | 1500 | —— | 1500 | —— |
| Mg | 264.9 | 370 | 231.82±1.64 | 370 | 231.82±1.64 | 370 | 231.82±1.64 | 330 | 143.59±1.02 |
| Fe | 21.0 | 18 | —— | 25 | 100.17±21.11 | 29 | 200.34±42.21 | 24 | 75.13±15.83 |
| Zn | 10.3 | 10.5 | 17.03±3.89 | 10.5 | 17.03±3.89 | 10.5 | 17.03±3.89 | 13 | 229.87±52.55 |
| Cu | 1.2 | 0.91 | —— | 0.9 | —— | 0.9 | —— | 1.5 | 52.14±13.39 |
| Mn | 4.8 | 4 | —— | 4 | —— | 4 | —— | 4.2 | —— |
| Se | 0.0416 | 0.065 | 1021.77±327.95 | 0.065 | 1021.77±327.95 | 0.065 | 1021.77±327.95 | 0.078 | 1589.43±510.15 |
| Cr | 0.1391 | 0.030 | —— | 0.033 | —— | 0.035 | —— | 0.035 | —— |
| Mo | 0.1610 | 0.025 | —— | 0.025 | —— | 0.025 | —— | 0.030 | —— |

Note: —— indicates that it is not advisable to obtain these elements through tea consumption.

**Table S8. Appropriate tea consumption to supplement elements for males aged 18 years and above based on average daily dietary intakes.**

| Element | Average daily normal intake （mg/d） | 18-29 years | | 30-49 years | | 50-64 years | | 65-74 years | | Over 75 years | |
|---|---|---|---|---|---|---|---|---|---|---|---|
| | | Recommended intake （mg/d） | Appropriate tea consumption （g/d） | Recommended intake （mg/d） | Appropriate tea consumption （g/d） | Recommended intake （mg/d） | Appropriate tea consumption （g/d） | Recommended intake （mg/d） | Appropriate tea consumption （g/d） | Recommended intake （mg/d） | Appropriate tea consumption （g/d） |
| Ca | 356.3 | 800 | 2628.08±582.11 | 800 | 2628.08±582.11 | 800 | 2628.08±582.11 | 800 | 2628.08±582.11 | 800 | 2628.08±582.11 |
| K | 1547.2 | 2000 | 2939.85±430.33 | 2000 | 2939.85±430.33 | 2000 | 2939.85±430.33 | 2000 | 2939.85±430.33 | 2000 | 2939.85±430.33 |
| Na | 6046.0 | 1500 | —— | 1500 | —— | 1500 | —— | 1400 | —— | 1400 | —— |
| Mg | 264.9 | 330 | 143.59±1.02 | 320 | 121.54±0.86 | 320 | 121.54±0.86 | 310 | 99.48±0.70 | 300 | 77.42±0.55 |
| Fe | 21.0 | 12 | —— | 12 | —— | 12 | —— | 12 | —— | 12 | —— |
| Zn | 10.3 | 12.5 | 187.30±42.82 | 12 | 144.73±33.09 | 12 | 144.73±33.09 | 12 | 144.73±33.09 | 12 | 144.73±33.09 |
| Cu | 1.2 | 0.8 | —— | 0.8 | —— | 0.8 | —— | 0.8 | —— | 0.7 | —— |
| Mn | 4.8 | 4.5 | —— | 4.5 | —— | 4.5 | —— | 4.5 | —— | 4.5 | —— |
| Se | 0.0416 | 0.060 | 803.45±257.88 | 0.060 | 803.45±257.88 | 0.060 | 803.45±257.88 | 0.060 | 803.45±257.88 | 0.060 | 803.45±257.88 |
| Cr | 0.1391 | 0.035 | —— | 0.035 | —— | 0.030 | —— | 0.030 | —— | 0.030 | —— |
| Mo | 0.1610 | 0.025 | —— | 0.025 | —— | 0.025 | —— | 0.025 | —— | 0.025 | —— |

Note: —— indicates that it is not advisable to obtain these elements through tea consumption.

**Table S9. Appropriate tea consumption to supplement elements for females aged 18 years and above based on average daily dietary intakes.**

| Element | Average daily normal intake（mg/d） | 18-29 years | | 30-49 years | | 50-64 years | | 65-74 years | | Over 75 years | |
|---|---|---|---|---|---|---|---|---|---|---|---|
| | | Recommended intake（mg/d） | Appropriate tea consumption（g/d） | Recommended intake（mg/d） | Appropriate tea consumption（g/d） | Recommended intake（mg/d） | Appropriate tea consumption（g/d） | Recommended intake（mg/d） | Appropriate tea consumption（g/d） | Recommended intake（mg/d） | Appropriate tea consumption（g/d） |
| Ca | 356.3 | 800 | 2628.08±582.11 | 800 | 2628.08±582.11 | 800 | 2628.08±582.11 | 800 | 2628.08±582.11 | 800 | 2628.08±582.11 |
| K | 1547.2 | 2000 | 2939.85±430.33 | 2000 | 2939.85±430.33 | 2000 | 2939.85±430.33 | 2000 | 2939.85±430.33 | 2000 | 2939.85±430.33 |
| Na | 6046.0 | 1500 | —— | 1500 | —— | 1500 | —— | 1400 | —— | 1400 | —— |
| Mg | 264.9 | 330 | 143.59±1.02 | 320 | 121.54±0.86 | 320 | 121.54±0.86 | 310 | 99.48±0.70 | 300 | 77.42±0.55 |
| Fe | 21.0 | 18 | —— | 18 | —— | 18 | —— | 10 | —— | 10 | —— |
| Zn | 10.3 | 8.5 | —— | 8.5 | —— | 8.5 | —— | 8.5 | —— | 8.5 | —— |
| Cu | 1.2 | 0.8 | —— | 0.8 | —— | 0.8 | —— | 0.8 | —— | 0.7 | —— |
| Mn | 4.8 | 4 | —— | 4 | —— | 4 | —— | 4 | —— | 4 | —— |
| Se | 0.0416 | 0.060 | 803.45±257.88 | 0.060 | 803.45±257.88 | 0.060 | 803.45±257.88 | 0.060 | 803.45±257.88 | 0.060 | 803.45±257.88 |
| Cr | 0.1391 | 0.030 | —— | 0.030 | —— | 0.025 | —— | 0.025 | —— | 0.025 | —— |
| Mo | 0.1610 | 0.025 | —— | 0.025 | —— | 0.025 | —— | 0.025 | —— | 0.025 | —— |

Note: —— indicates that it is not advisable to obtain these elements through tea consumption.
